# Effects of the COVID-19 Pandemic on Mental Health in Peru: Psychological Distress

**DOI:** 10.3390/healthcare9060691

**Published:** 2021-06-08

**Authors:** Carlos Ruiz-Frutos, Juan Carlos Palomino-Baldeón, Mónica Ortega-Moreno, María del Carmen Villavicencio-Guardia, Adriano Dias, João Marcos Bernardes, Juan Gómez-Salgado

**Affiliations:** 1 Department of Sociology, Social Work and Public Health, Faculty of Labour Sciences, University of Huelva, 21007 Huelva, Spain; frutos@uhu.es; 2 Safety and Health Postgraduate Programme, Universidad Espíritu Santo, Guayaquil 092301, Ecuador; 3 Faculty of Health Sciences, Universidad Científica del Sur, Lima 15054, Peru; jpalomino@cientifica.edu.pe; 4 Department of Economy, Faculty of Labour Sciences, University of Huelva, 21007 Huelva, Spain; 5 Faculty of Nursing, Universidad Hermilio Valdizan, Huánuco 10000, Peru; mvillavicencio@unheval.edu.pe; 6 Public (Collective) Health Grade Program, Botucatu Medical School, Paulista State University/UNESP, Botucatu, Sao Paulo 18618687, Brazil; dias.adriano@unesp.br (A.D.); jmbernardes@yahoo.com (J.M.B.)

**Keywords:** COVID-19, psychological distress, preventive measures, mental health, Peru

## Abstract

This pandemic has been classified as a “psychological pandemic” that produces anxiety, depression, post-traumatic stress disorder, and sleep disorders. As the mental health effects of the Coronavirus Disease 2019 (COVID-19), caused by SARS-CoV-2, continue to unfold, there are still large knowledge gaps about the variables that predispose individuals to, or protect individuals against the disease. However, there are few publications on the effects of the COVID-19 pandemic on the mental health of citizens in Latin American countries. In this study, the effects that COVID-19 had on citizens of Peru have been described. For this, 1699 questionnaires, collected between 2 April and 2 September 2020, were analyzed. Descriptive, bivariate analysis was performed with odds ratio (OR) calculations and a data mining methodology. Sociodemographic variables (from the General Health Questionnaire), health conditions and perception, symptoms, and variables related to contact and preventive measures regarding COVID-19 were analyzed. As compared to other countries, less affectation of mental health and increased use of preventive measures were observed. It has been suggested that the country’s precarious health system and poverty rates prior to the pandemic may justify higher mortality figures in Peru than in other Latin American countries, despite prompt action for its containment and compliance with the protective measures. Psychological distress had a greater incidence in women, young people, people without a partner, and people without university studies. The most significant conditioning variables were self-perceived health status, headache or muscle pain over the past 14 days, level of studies, and age. The extensive use of preventive measures against COVID-19 is in line with the strict legislative measures taken, and this is, in turn, in line with other countries when looking at the lower effect on mental health, but contrary when focusing on the high lethality identified. The need to include the economy or availability and quality of healthcare in future studies arises, as well as the suitability to analyze the cause for differences between countries.

## 1. Introduction

At the end of 2019, in Wuhan City, a group of 44 people presented pneumonia of unknown origin; eleven days later, the first case of death caused by a new coronavirus was reported [1]. On 31 December 2019, the WHO declared the first case, but it is suspected that it had already been detected on 17 November [2]. This infectious disease spread rapidly, and, on 21 January 2020, the virus reached the American continent, with the first case reported in the USA. Its spread continued, and, on 12 March, the WHO declared the status of international pandemic [3]. Latin America has overcome major recent health challenges such as Zika, but they were not as lethal as the current COVID-19, the Coronavirus Disease 2019 caused by SARS-CoV-2, with high rates of subclinical infections and inconsistent and insufficient diagnostic tests detected, making it difficult to know the actual number of people infected [4]. Analyzing deaths during 2020 in Peru, Ecuador, Mexico, and Spain, there were significantly greater excess deaths than the ones recorded for COVID-19, something that has not happened in Denmark, Germany, or Norway [5]. By the end of February 2020, Brazil became the first Latin American country to report the first case of COVID-19, followed by Ecuador, Argentina, and Chile. On 6 March 2020, Peru reported its first case, and nine days later, the Temporary National State of Emergency started, which included social distancing and the closure of borders [6,7,8]. On 26 March 2021, Peru ranked 20th globally in the number of confirmed cases (1,492,519 cases) and 4th in Latin America, after Colombia, Argentina, and Mexico; regarding lethality, it was ranked 15th globally (50,656 deaths) and 4th in Latin America, having administered 718,881 doses of vaccine [9,10].

In March 2020, the United Nations warned of the effects of the pandemic on the most vulnerable segments of the population, with food distribution effects, difficulty complying with hygiene measures, and lack of access to water or teaching, the latter conditioned by the unavailability of resources to adapt to online training. Gender-based violence and the ease of losing jobs also increased, as well as risk of contagion among groups with fewer economic resources. A set of policies have been developed to help respond to the economic and social challenges provoked by the impact of COVID-19 [11].

Peru declared the first state of emergency for 15 days, including a national lockdown [12], which was extended over time, providing exceptional and temporary measures to prevent the spread and effects of the pandemic, creating a network to support the highest-risk adult population or people with severe disabilities, creating bonuses for healthcare staff, funding biosecurity actions, conditioning and enabling spaces to strengthen the provision of healthcare services, enhancing emergency telephone services, collecting samples at home or teleworking, and increasing specialized hospital capacity [12,13]. Although economic measures were put in place in Peru for lower-income people, making it easier for them to be confined at home, fewer than 15.7% received them. This situation is explained by not having information about vulnerable people and because 59.8% of the adult population did not have a bank account to receive their welfare assistance money [14]. Peru, being one of the first countries in Latin America where its government took steps to control the pandemic and whose inhabitants were quite willing to comply, could not avoid generating the highest mortality rates from COVID-19, something that can be understood by Peru having the lowest per capita income, higher poverty rates, and a worse health system, as compared to other Latin American countries [15].

In response to the increased contagion among essential activity workers, who had not been subject to cessation of their activity, in November 2020, the Ministry of Health issued a resolution to protocolize the actions of occupational healthcare and safety services of companies for the management of COVID-19 [16]. Measures were put in place for the cleaning and disinfection of workspaces, washing and disinfection of hands, and raising awareness for the prevention of contagion in the workplace and personal protection, as well as for the monitoring of workers returning to work through guidelines [8,16]. Differences in sex and age variables have been found regarding the degree of compliance with preventive measures against the COVID-19 contagion among Latin American countries [17], as in other Hispanic countries outside Latin America [18].

During the beginning of 2021, there has been a noticeable increase in cases, demonstrating that two out of three cases were over 60 years old and with more lethal results (14.7%), with a higher prevalence of cases in men [13]. A percentage of infected people remain asymptomatic, but those with symptoms include fever, cough, dyspnea, sore throat, nasal congestion, nausea, vomiting, diarrhea, fatigue, myalgias, chills, dizziness, headache, anosmia, and ageusia [19,20]. On the other hand, asymptomatic cases are also known to be an important source of contagion, and fever is the most common symptom of patients with severe and non-severe disease [21]. The sequelae that have been described are muscle fatigue and weakness [22], apart from the well-known lung effect, causing interstitial pneumonitis and severe acute respiratory distress syndrome (ARDS), that also affect multiple organs, particularly the cardiovascular system [23,24].

This pandemic has been classified as a “psychological pandemic” that produces anxiety, depression, posttraumatic stress disorder, and sleep disorders, which most affect frontline healthcare workers, migrants, and workers in contact with the public [25]. The psychological effects for these groups are known from previous epidemics, which can also cause physical effects such as headaches or gastric disorders [26]. Infected people show higher levels of anxiety, depression, stress, intrusions, hyper-vigilance, and avoidance [27], as well as higher levels among those with previous physical or mental health problems [28].

The phenomenology and psychopathology derived from the COVID-19 pandemic appear to differ from those resulting from natural disasters such as a hurricane. The first is reactive in nature, for fear of contagion, confinement to reduce transmission, or financial hardship produce feelings of sadness, fear, anger, paranoia, depression, anxiety, and somatization. Unlike this, the second situation generates post-traumatic stress. Short-term adjustment issues and long-term adaptation to the uncertain future may be reasonable or expected responses [29].

The arrival of this disease in Peru has caused a lifestyle change as an effect of complying with quarantine and social distancing, with the consequent impact on mental health. Among the effects, insomnia, anxiety, and depression have been described [22,30], as well as psychiatric disorders and even alopecia in women [24]. Increases in the prevalence of depression were observed, from 30.5% to 31–36%, and for anxiety disorders from 22.4% to 30–35% [31]. The Ministry of Health established, in June 2020, a Mental Health Plan that, supported by scientific knowledge on the effects of the pandemic on mental health and specific studies conducted in the country, fostered psychosocial support tools such as the creation of a specific telephone line [32]. The young population has been found to have increased anxiety, depression, and feelings of uncertainty, manifestations of psychological distress that can be explained by lockdown conditions and intolerance to uncertainty [33]. The illness or death of a family member or friend and the economic impact are other variables that enhance the effects of the pandemic on mental health [25], as well as the effects on people who already suffer from mental illnesses [34].

Healthcare professionals represent the group that is the subject of most research on the effects of COVID-19. This may be explained by the fact that the situation experienced causes them stress, tiredness, frustration, and isolation, apart from the anxiety, depression, and mental effects that are common among the general population [25]. In Peru, it has been found that, among physicians, labor stress appears to have been associated with the lack of protective equipment, lack of health resources, and/or high workload [35]. The health authority of Peru has suggested that non-specialist healthcare professionals in psychiatry could also identify psychiatric problems in the early pandemic periods in order to prevent mental health disturbances, for which a technical guide was published, generating a space of trust between the patient and the healthcare worker [36]. Among physician students in Peru, high levels of anxiety (75.4%) were found, mostly in the female sex [37], demonstrating the negative effects of virtual teaching forced by the pandemic and the greater impact it has had on groups with fewer technological resources [38].

There are few publications on the effects of the COVID-19 pandemic on the mental health of citizens in Latin American countries. Previous publications have found that the COVID-19 pandemic has not affected all countries equally, and this may also result in a different impact on the mental health of the population. Thus, the expected number of deaths or people who would recover from the disease in Peru during the 60 days prior to 21 September 2020 (60,000 and 475,000, respectively) differed from other countries in Latin America and other parts of the world. Peru, similar to other countries, has shown an exponential increase in tendencies [39]. It has been suggested that the country’s precarious health system and poverty rates prior to the pandemic may justify higher mortality figures in Peru than in other Latin American countries, despite prompt action for its containment and with Peruvian citizens being more compliant with the measures imposed by the government than other Latin American populations [4,15].

Crossing the levels of psychological distress with sociodemographic variables, perception of health, presence of physical symptoms, history of contact with contaminated people or objects, and preventive measures adopted can help us to identify those variables or groups where it is more efficient to adopt preventive measures. This study presents data on the effects of COVID-19 on the mental health of citizens in Peru, especially in the development of psychological distress.

## 2. Materials and Methods

### 2.1. Design and Sample

Cross-sectional observational study

The total number of questionnaires analyzed was 1699, collected between 2 April and 2 September 2020. The inclusion criteria were: being 18 years of age or older, residing in Peru during the pandemic, and accepting the informed consent. Questionnaires were received from all 25 departments into which the country of Peru is divided, highlighting the departments of Lima (38.14%), Huanuco (12.77%), and Ancash (10.59%).

### 2.2. Instruments and Data Collection

The original questionnaire was validated for the Spanish population, adapting questions from previous studies [40] and using a review of the literature on publications of previous epidemics [41]. To shorten the validation time and be able to collect data at the start of the pandemic, previously validated instruments were included. The questionnaire was assessed by a panel of experts, consisting of physicians, nurses, psychologists, epidemiologists, and public health experts. No comprehension problems were found, with a Cronbach’s alpha coefficient of 0.86 and good psychometric properties obtained through a pilot test with 57 people of different age, sex, educational level, profession, and geographical area. Subsequently, the questionnaire was culturally and linguistically adapted to the Peruvian population, modifying questions which may be difficult to understand.

The questionnaire included sociodemographic data: age, sex, level of studies, cohabitation status, employment status, parental status (i.e., having children or not), pet or disability status, and confinement status (i.e., strict, except purchase or work; not in confinement; or in another situation).

Mental health and psychological well-being were measured using the Goldberg’s General Health questionnaire (GHQ-12) [42]. It is a 12-item questionnaire with four answer options, assigning 0 points to the first two and 1 point to the last two, with a total score ranging from 0 to 12. The set cut-off point for the general population was 3, considering psychological distress for those with scores greater than or equal to 3.

Perceived symptoms were collected over the past 14 days: sore throat, headache, rhinitis, cough, fever, chills, myalgia, dizziness, diarrhea, or shortness of breath, which are the most common physical symptoms associated with COVID-19 according to information published by the World Health Organization. They were asked whether they had a chronic illness, took medication at the time of answering the questionnaire, had been hospitalized, or had required medical attention in the last 14 days.

A history of contact was obtained during the previous 14 days through three items: possible contact (more than 15 min less than two meters away), casual contact with confirmed infected persons, or contact with people or materials suspected of being infected, as well as the existence of an infected family member diagnosed through diagnostic testing. Their self-perceived health status over the last two weeks was measured with five response levels, from lousy to optimal, grouped for the final analysis into two categories, this being a well-known good indicator for predicting mortality [43].

The prevention measures carried out were measured with five response options, categorized from never to always, with respect to the frequency with which they were performed: covering the mouth with the elbow when coughing or sneezing; avoiding sharing utensils (e.g., fork) during meals; washing hands with soap and water; washing hands with hydroalcoholic solution; washing hands immediately after coughing, touching the nose, or sneezing; washing hands after touching potentially contaminated objects; wearing a mask regardless of the presence of symptoms; keeping at least a meter-and-a-half distance from others.

### 2.3. Procedure

It is an international research project coordinated by a group of researchers in Spain, where the original instruments were validated. It takes place in 16 countries, with a majority of Latin American countries, at different stages of adaptation and implementation and using the same methodology except for the different adaptations to each country or the dates of data collection. For this specific study, an occupational physician from a Peruvian university and an expert researcher coordinated the project in Peru, assisted by researchers from other universities from different geographical areas in order to facilitate its dissemination.

The Qualtrics storage and survey platform was used to collect information through an online questionnaire. The same methodology of the European study on Living, Working and COVID-19 by Eurofound [44] was chosen for sampling, as well as the non-probabilistic sampling method: snowballing method. The collaboration of scientific societies and universities was requested, using social networks and press for dissemination. There were no incentives to participate in the study.

### 2.4. Data Analysis

Absolute and relative frequencies are provided for each of the variables under study, as well as the percentages per row associated with the psychological distress variable in the contingency table. The bivariate analysis allowed for the determination of both which variables are related and the Odds Ratio (OR), along with the associated confidence interval.

The chi-squared automatic interaction detection (CHAID) method identified which variables are most related to the classification criterion using the chi-squared independence test and selecting the most significant factor. With this method, the sample is divided according to the levels of the chosen factor, and each resulting group is reapplied the same criterion; after that, the sample is divided repeatedly until it is not possible to continue doing so or until no other significant factor is found.

All analyses were carried out with the SPSS 26.0 statistical software (IBM, Armonk, NY, USA).

### 2.5. Ethical Principles

The ethical principles set out in the Declaration of Helsinki have been followed. The participants’ permission was obtained through a written informed consent in which they expressed their voluntary desire to participate in the study. Data were recorded anonymously and treated confidentially. The study has been authorized by the Ethics Committee of the Universidad Científica del Sur, in Peru (Constancia No. 083-CEI-CIENTIFICA-2020), and in Spain by the Research Committee of Huelva, belonging to the Regional Ministry of Health of Andalusia (PI 036/20).

## 3. Results

### 3.1. Sociodemographic Data

The mean age of responders was 40.85 years (SD = 13.97), with an age of less than or equal to 40 years in 51.5% of cases. In the sample, 56.2% were women, with a mean age of 38.50 (SD = 13.18), with men being 43.97 (SD = 14.39). In the sample, 85.9% had a university educational level or higher. With regard to the employment situation, the highest percentage corresponded to public employees (34.1%), followed by workers from private companies (23.0%) and the self-employed (7.0%). No significant differences were found regarding the number of responses for people living or not as a couple (51.7% did not), having children (57.4% had them), or having a pet (55.2% had one). A small percentage claimed to have some degree of disability (5.1%). In the sample, 38.0% of cases were strictly confined; 56.4% were confined except for work and purchases; 2.5% were not confined; and 3.1% were in other situations (Table 1).

### 3.2. Psychological Distress in the Sample Studied

With a cut-off point of GHQ-12 ≥ 3, 59.7% of globally studied cases have psychological distress. The overall mean score on the 12 items (GHQ-12) is M = 4.18, SD = 3.52, and the questionnaire has an optimal associated measurement scale reliability coefficient (Cronbach’s α = 0.886).

The items with the highest scores have been: “Have you felt constantly overwhelmed and in tension?” (M = 2.64; SD = 0.92); “Have you been able to enjoy your normal daily activities?” (M = 2.60; SD = 0.86); and “Have your concerns made you lose a lot of sleep?” (M = 2.51; SD = 0.96). On the other hand, the items with the lowest scores have been: “Have you thought that you are a worthless person?” (M = 1.28; SD = 0.67) and “Have you lost confidence in yourself?” (M = 1.69; SD = 0.91) (Table 2).

### 3.3. Sociodemographic Data and Psychological Distress

The percentage of women with psychological distress (64.9%) is higher than that of men (53.0%), *p* < 0.001, Odds Ratio (OR) = 0.608, 95% CI (0.500, 0.626). Moreover, psychological distress is higher among those who are 40 years of age or younger (65.7%) than among the older individuals (53.4%), *p* < 0.001, OR = 1.667, 95% CI (1.370, 2.029); among those who do not have a partner (63.4%), compared to those who do have one (55.7%), *p* < 0.001, OR = 1.374, 95% CI (1.131, 1.669); those without children (65.9%), compared to those who have them (55.1%), *p* < 0.001, OR = 0.635, 95% CI (0.520, 0.775). The highest psychological distress was found among participants with non-university educational level (68.2%) as compared to those with university level or higher (58.3%), *p* = 0.004, OR = 1.535, 95% CI (1.147, 2.054). Significant differences are also seen according to the confinement variable (*p* = 0.029), where the percentage of cases with higher psychological distress is found in people in strict confinement (62.2%), followed by those in confinement except for purchase or work (59.1%) and those in another situation (53.8%). In the case of non-confinement, the number of cases without psychological distress (59.5%) is higher. On the contrary, there are no differences in the generation of psychological distress when having a pet or not or having a disability or not, or according to the type of work, specifically, working as a public employee, working in a private company, or being self-employed (Table 1).

### 3.4. Physical Symptoms, Health-Related Variables, and Psychological Distress

The mean number of symptoms in the last 14 days was 1.71 (SD = 1.80), the most common being: headache (38.1%), nasal congestion (27.4%), muscle pain (25.0%), and sore throat (24.4%).

There is an observed difference in psychological distress when comparing having a symptom or not, for all symptoms, *p* < 0.001. Having a fever is highlighted, with OR = 5.024, 95% CI (2.264, 11.147); as well as having breathing difficulties, OR = 2.848, 95% CI (1.727, 4.695); dizziness, OR = 2.588, 95% CI (1.787, 3.745); headache, OR = 2.509, 95% CI (2.031, 3.098); and muscle pain, OR = 2.392, 95% CI (1.874, 3.054). There is also a difference in psychological distress with all other symptoms, from highest to lowest: cough, chills, cough, diarrhea, sore throat, and nasal congestion (Table 3).

In the sample, 28.8% claimed to have a chronic illness, and 27.4% were taking medication. By contrast, only 5.3% had required medical care during the last 14 days and 0.5% hospitalization. There has been a statistically significant difference regarding developing psychological distress when taking medicines (65.2%) and not doing so (57.6%), *p* = 0.005, OR = 1.376, 95% CI (1.102, 1.717), as well as when needing medical attention in the last 14 days (74.4%), as compared to not requiring it (58.9%), *p* = 0.003, OR = 2.036, 95% CI (1.255, 3.304) (Table 3).

In the sample, 75.3% claimed to have an optimal self-perceived health, with statistically significant differences in psychological distress among those reporting optimal health (52.8%) and those who perceived it as mediocre or lousy (80.7%), *p* < 0.001, OR = 0.268, 95% CI (0.206, 0.350) (Table 3).

### 3.5. Contact History and Psychological Distress

In the sample, 33.4% had previous contact with an infected person, or claimed not knowing if they ha, for more than 15 min and with less than 2 meters distance, compared to the remaining 66.6% who claimed they had not been in such a situation. For 38.7% who had casual contact with an infected person or were not aware if they had, 43.3% had contact with a person or material suspected of being infected or did not realize it, and 19.6% had contact with an infected relative or did not know if they had. The percentage of participants with the diagnostic test conducted was 13.1% (Table 4).

There is a statistically significant association between having psychological distress and the following variables: contact with an infected person for more than 15 min and less than 2 meters distance, *p* = 0.018, OR = 1.285, 95% CI (1.044, 1.582); casual contact with an infected person, *p* = 0.015, OR = 1.282, 95% CI (1.049, 1.582); contact with a person or material suspected of being infected, *p* = 0.0.38, OR = 1.230, 95% CI (1.011, 1.498); and status of having an infected relative, *p* = 0.001, OR = 1.524, 95% CI (1.183, 1.963). On the contrary, there has been no statistically significant association between having psychological distress and having had performed the diagnostic test (Table 4).

### 3.6. Preventive Measures and Psychological Distress

The preventive measure with a higher mean score was “Washing hands with soap and water” (M = 4.77; SD = 0.50), followed by “Washing hands after touching potentially contaminated objects” (M = 4.62; SD = 0.68). On a second level, the following measures are “Covering mouth” (M = 4.52; SD = 0.73), “Keeping at least one and a half metres away” (M = 4.51; SD = 0.71), and “Wearing a mask regardless of the presence of symptoms” (M = 4.48; SD = 0.93).

On the contrary, preventive measures with lower scores, but with greater variability, were: “Avoiding sharing utensils” (M = 3.96; SD = 1.30); “Washing hands with hydroalcoholic solution” (M = 4.00; SD = 1.03); and “Washing hands after coughing, touching the nose, or sneezing” (M = 4.06; SD = 1.02) (Table 5).

A statistically significant association has been found between having psychological distress and the use of the following preventive measures: “Washing hands after coughing, touching the nose, or sneezing”, *p* < 0.001; “Keeping at least a metre and a half distance”, *p* = 0.017; “Covering the mouth” *p* = 0.021; and “Washing hands with soap and water”, *p* = 0.035 (Table 5).

### 3.7. Segmentation Tree Displaying the Level of Psychological Distress

The segmentation tree for the level of psychological distress is conditioned by self-perceived health over the last 14 days, with 52.8% (676) of participants for whom the perception was optimal versus 80.7% (338) for whom the perception was mediocre or lousy. In turn, for those who had an optimal self-perceived health, psychological distress was conditioned by having had or not had a headache during the last two weeks, being 46.8% (406) those who had not, and 65.4% (270) those who had. Among the former, psychological distress was conditioned based on having had muscle pain during the last two weeks, with 77.1% (101) reporting muscle pain, and 59.9% (169) not having had such pain. Among those who had not had a headache during the last two weeks, psychological distress was conditioned by being over 40 years old, 40.9% (201), or 40 years old or younger, with psychological distress present in 54.7% (205) of them.

**Figure 1:**
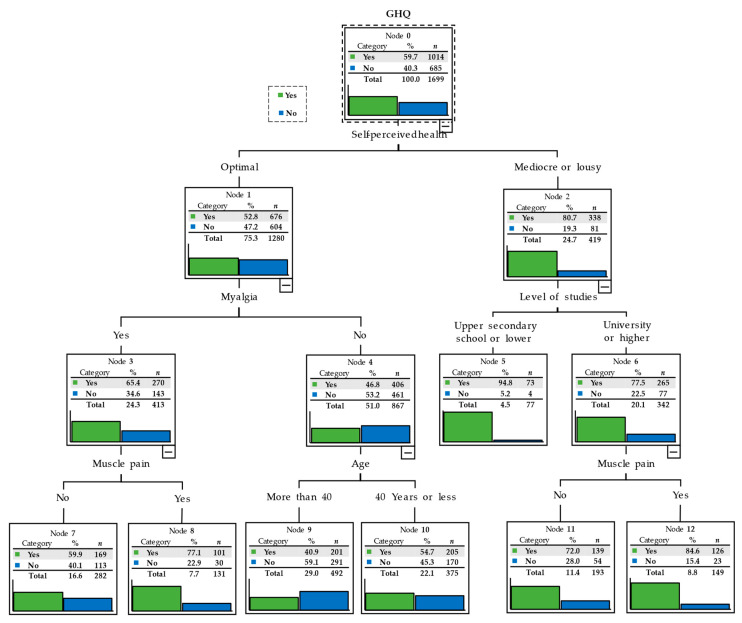
Segmentation tree displaying the level of psychological distress.

The most significant variable for generating psychological distress, among those participants with mediocre or poor self-perceived health, was the level of studies, i.e., 77.5% (265) had a university or higher educational level, and 94.8% (73) of those had no university studies. Among participants with university or higher-level studies, psychological distress was mediated based on having had muscle pain during the last two weeks, with psychological distress being present in 72.0% (139) of those who reported such pain, and 84.6% (126) of those who had not had muscle pain during the last two weeks (Figure 1).

## 4. Discussion

The aim of this study was to analyze the effects of COVID-19 on the mental health of citizens in Peru, especially in the development of psychological distress. In this way, a 59.7% of the people had high psychological distress (GHQ-12 ≥ 3), a lower value than the one observed in Spain (72.0%) [18]. The percentage of women’s participation (56.2%) was lower here than in the study carried out in Spain, with psychological distress was also lower in women in Peru than in Spain, and, similar to most countries investigated, higher psychological distress was found among women than in men [17,18]. It is known that sex is a variable that influences the level of COVID-19 contagion and, in a study on people assisted with COVID-19 conducted in eight Latin American countries, Peru presented a higher percentage of women [4]. In the present study, higher psychological distress has also been found among younger people, but, on the contrary, higher psychological distress was found among those with the lowest level of education. Unlike in Peru, where the percentage of people with psychological distress was higher among those who did not have children (65.9%, compared to 55.1% of those who do have children), in the study carried out in Spain, the percentage of people with psychological distress was higher among those who had children 73.9%, compared to those who did not, 70.2% [18].

We have seen how the symptoms described regarding the physical part of the illness differ in their frequency from those found in another study in Spain [18]. Specifically, fever was indicated in 38.1% of the current sample, compared to 53.3% in Spain, and cough in 17.5%, compared to 30.6%. On the contrary, coryza was more frequent in this study in Peru (27.4%) than in the research carried out in Spain (21.0%).

Stress has been more likely to happen in women and young people in Spain, Ecuador, Colombia, and Chile [17,18,45]. Increased stress in young people could be driven by the impact of the pandemic on their work, education, and social life situation and, to a lesser extent, by their perception of health risk, which is more frequent in older people. Young males perceive the situation worse than young women, while older women perceive the situation worse than the men of the same age group. Older women also comply with preventive measures to a greater extent than men [17]. Differences have been observed in the data between countries (Colombia, Ecuador, Chile, and Spain), with greater stress in young people and people with a higher educational level [25].

The effects on mental health could be related to the legislative measures undertaken to prevent the contagion or evolution of the pandemic. Moreover, the level of compliance and differences between population groups according to socioeconomic, educational, and working conditions are variables that are known to determine physical and mental health. This requires reliable sources of information, something that has been highly questioned as seen in previous studies [4].

This study showed very high percentages on the use of preventive measures, which contrasts with the high mortality rates [9]. Moreover, recent research has proved that entitled workers were positively associated with failure to follow instructions and little concern about virus-mitigating behaviors in the fight against COVID-19 [46]. Therefore, more important than knowing preventive measures established by the country authorities would be to know the level of compliance, given the difficulty to compare results between different countries if the same measures have not been used. It has been estimated that 1.7 million lives could have been saved in the United States if handwashing had been practiced frequently, massive meetings cancelled, and a safe space, with social distancing, had been maintained in the early stages of the pandemic [47]. The most valued preventive measure, both in this study and in the one in Spain, is “Washing hands with soap and water”, followed by “Washing hands after touching potentially contaminated objects”. A difference was found between “Wearing a mask regardless of the presence of symptoms”, which was much higher in Peru, M = 4.48 (SD = 0.93), than in Spain, M = 3.12 (SD = 1.53) [48], which can be explained by the fact that study data in Spain were collected much earlier and by the changes that have been experienced in the level of information during this time. In the present study, the assessment of the use of certain preventive measures was associated with the level of psychological distress, although it did not occur with the use of a mask regardless of the presence of symptoms.

Optimal self-perceived health was reported by 75.3% of participants, and that was associated with the development of psychological distress, as in previous studies [49]. A study developed in 28 European countries found that men are more optimistic than women about the pandemic and that this difference increased over time [44]. Precisely, this optimism can be related to the optimal perception of the health status, observing in our study that an optimal perception is associated with lower psychological distress, something that is not uncommon since it is well known that self-perceived health is a good predictor of mortality [43].

It has been proposed to implement sex-sensitive preventive strategies [17,50,51], also related to age [17,18], socioeconomic level [14], culture, and ethnicity [52]. In Colombia, Ecuador, and Spain, it was seen that woman and the elderly were most involved with self-care activities and healthy routines, while in Chile, it was men and those under 60 years of age who had higher levels of healthy routines and compliance with published guidelines for prevention [17].

The pandemic can serve to strengthen health systems and health promotion programs [17,53,54,55]. In this line, healthcare resources could be particularly scarce during a serious pandemic situation. However, timely interventions such as providing psychological support could restore mental wellness and reduce the consequences of isolation and loneliness on the population. Telemedicine and informal support groups have been particularly useful in combating mental disorders that could be influenced by over-information, misinformation or the existence of fake news [56]. It is known that cultural determinants play an important role in controlling the behavior of infectious pandemics, as happens with COVID-19, as well as the importance of including cultural values in public health interventions [57] and the existence of inequalities in health care associated with the ethnicity variable in Latin American countries [58]. Previous studies have positively associated neuroticism, extroversion, and kindness with the perceived cost of complying with preventive measures, while awareness showed a negative association [59]. It has been proposed that, in countries where there are no reliable indicators to measure the number of people infected and dead by COVID-19, as is the case in many Latin American countries, the relative excess of deaths over the time period should be used as a more reliable indicator of the development of the disease [5].

Though this may deviate from the theoretical objectives of the study, some of the health effects of the COVID-19 pandemic are as diverse as its environmental impact, derived from the use of non-biodegradable protective measures, as seen off the coast of Lima [60]. This is just one example of variables that can condition the different effects of the pandemic on the population, depending on the level of environmental concern or awareness of the country and with regard to some groups such as the youth, just as the educational and socioeconomic level or sex influence the level of psychological distress produced during the COVID-19 pandemic.

A limitation of this study, similar to others implemented in the country [14], is that only people with internet access could participate, leaving out groups of lower income and educational level, with a presumably higher risk of contagion and with a risk perception different from the analyzed sample. This possible bias is visible in that 85.9% of those who participated had university or a higher educational level, far from the average in the country and explained by the dissemination method conducted by researchers in universities. Other limitations are the lack of benchmark indices on psychopathology and a possible selection bias (of unknown extent because attrition cannot be evaluated). One last limitation could be related to the measurement of self-reported COVID-19 prevention, as the instrument for this study has been created and validated ad hoc. In this way, it could be relevant to use well-validated scales in future studies [61]

On the contrary, one positive relevant aspect to note is the use of the same measurement instrument, validated in Spain and adapted to other countries in Latin America and Europe, since it facilitates the comparison of the data.

In Latin America, in the first phase of the pandemic, COVID-19 diagnostic methods were developed and used in a short period of time and with many limitations, which generated wrong results and insufficient diagnosis, thus increasing the level of uncertainty and making it difficult to estimate the total number of cases, and even to initiate control measures, resource planning, or benchmarking with neighboring countries [4]. However, over time, new tools have been developed for a rapid identification of the infected that allow their isolation and treatment, aiming to enhance their use through artificial intelligence and machine learning that integrate clinical and laboratory data [62]. This, together with vaccination, should reduce uncertainty and thereby effects on mental health, although tiredness from restrictive measures maintained over time plays the opposite role by increasing the negative effects on mental health.

A hypothesis that could be analyzed in future studies is whether the cause of a lower level of psychological distress among Peruvian women can be explained by greater family support or even pregnancy, which has proved to increase protection against COVID-19 mental health effects by diminishing depressive symptoms and increasing resilience and positive thinking [63]. Future lines of research have been proposed, including new variables related to the economy or health system, as well as to understanding the evolution of mental health effects over time. A good initiative is the study initiated in Thailand on healthcare staff and the general population [64]. With regard to the study at hand, it is planned to re-measure the results after the pandemic progresses and compare them with the effects in the first phase (data from this study). A short questionnaire has been validated in the Spanish population that measures fear of and anxiety to COVID-19 [65], which is planned to be implemented in Peru and other Latin American countries, once adapted.

## 5. Conclusions

Women and young people showed higher psychological distress related to COVID-19, as seen in the literature. The most significant variables that condition the level of psychological distress were self-perceived health, headache or muscle pain during the last 14 days, level of studies, and age. Fever and cough have been less frequent as compared to the population in Spain. However, some results on the effects of the COVID-19 pandemic on mental health have been found to differ from those found in other Latin American countries. Certain preventive measures such as handwashing or the use of a face mask have been highly identified in Peru, which can be explained by the moment of the data collection, the educational level, and the socio-labor or cultural circumstances of the sample. However, this high presence of measures to prevent contagion contrasts with the high COVID-19 lethality experienced in Peru, as compared to other Latin American countries, which again could be explained by the high educational level of the sample (well above the country’s average), and by the characteristics of the public health system and the socioeconomic management of this country. It would be beneficial if other variables could be included in future studies, such as socioeconomic factors and the availability and quality of healthcare, so as to analyze their influence on mental health and the use of the proposed measures to prevent contagion.

## Figures and Tables

**Table 1:** Association between sociodemographic variables and psychological distress during the pandemic.

GHQ Total (*n* = 1699)						
	*N* (%)	Yes (*n* = 1014)	No (*n* = 685)	χ^2^	*p*	Odds Ratio (Confidence Interval at the 95 Level)
Sex						
Male	744 (43.8)	53.0	47.0	24.879	<0.001	0.608
Female	955 (56.2)	64.9	35.1			(0.500, 0.740)
Age *						
40 years old or younger	868 (51.5)	65.7	34.3	26.186	<0.001	1.667
Older than 40	816 (48.5)	53.4	46.6			(1.370, 2.029)
Marital status						
Without a partner	879 (51.7)	63.4	36.6	10.279	0.001	1.374
With a partner	820 (48.3)	55.7	44.3			(1.131, 1.669)
Level of studies						
Upper secondary school or lower	239 (14.1)	68.2	31.8	8.388	0.004	1.535
University or higher	1460 (85.9)	58.3	41.7			(1.147, 2.054)
You are **						
Self-employed	119 (7.0)	58.0	42.0	1.541	0.463	
Public worker	579 (34.1)	55.4	44.6			
Private-company worker	390 (23.0)	52.3	47.7			
Children						
Yes	975 (57.4)	55.1	44.9	20.166	<0.001	0.635
No	724 (42.6)	65.9	34.1			(0.520, 0.775)
Pet						
Yes	937 (55.2)	59.7	40.3	0.000	0.982	1.002
No	762 (44.9)	59.7	40.3			(0.825, 1.218)
Disability						
Yes	86 (5.1)	69.8	30.2	3.829	0.050	1.594
No	1613 (94.9)	59.1	40.9			(0.996, 2.553)
Confinement						
Strict	646 (38.0)	62.2	37.8	9.040	0.029	
Except purchase or work	959 (56.4)	59.1	40.9			
No	42 (2.5%)	40.5	59.5			
Other situation	52 (3.1%)	53.8	46.2			

*—Grouped variable from the median value. * (*n* = 1684); ** (*n* = 1088).

**Table 2:** Psychological Distress: General Health Questionnaire GHQ-12.

	Total (*n* = 1699)
Item	M (SD)
1. Have you been able to properly concentrate on what you were doing?	2.48 (0.73)
2. Have your worries made you lose a lot of sleep?	2.51 (0.96)
3. Have you felt you are developing a relevant role in life?	1.94 (0.86)
4. Have you felt capable of making decisions?	1.98 (0.78)
5. Have you felt constantly overwhelmed and stressed?	2.64 (0.92)
6. Have you felt unable to overcome your difficulties?	2.10 (0.91)
7. Have you been able to develop your normal daily activities?	2.60 (0.86)
8. Have you been able to properly face your difficulties?	2.20 (0.73)
9. Have you felt unhappy or depressed?	2.26 (0.98)
10. Have you lost confidence in yourself?	1.69 (0.91)
11. Have you thought that you are a worthless person?	1.28 (0.67)
12. Do you feel reasonably happy given the circumstances?	2.10 (0.77)
GHQ-12 (Score on a scale of 12)	4.18 (3.52)
Cut-off point ≥ 3	*n* (%)
Yes	1014 (59.7)
No	685 (40.3)

**Table 3:** Association between physical symptoms and current health status with psychological distress during the pandemic.

	Total (*n* = 1699)					
		GHQ				
	*N* (%)	Yes (*n* = 1014)	No (*n* = 685)	χ^2^	*p*	Odds Ratio (Confidence Interval at the 95 Level)
Physical Symptoms						
Fever						
Yes	57 (3.4)	87.7	12.3	19.267	<0.001	5.024
No	1642 (96.6)	58.7	41.3			(2.264, 11,147)
Cough						
Yes	297 (17.5)	71.7	28.3	21.665	<0.001	1.903
No	1402 (82.5)	57.1	42.9			(1.447, 2.502)
Myalgia						
Yes	647 (38.1)	72.8	27.2	74.697	<0.001	2.509
No	1052 (61.9)	51.6	48.4			(2.031, 3.098)
Muscle pain						
Yes	425 (25.0)	74.4	25.6	50.696	<0.001	2.392
No	1274 (75.0)	54.8	45.2			(1.874, 3.054)
Dizziness						
Yes	176 (10.4)	77.8	22.2	26.905	<0.001	2.588
No	1523 (89.6)	57.6	42.4			(1.787, 3.746)
Diarrhea						
Yes	198 (11.7)	72.2	27.8	14.647	<0.001	1.881
No	1501 (88.3)	58.0	42.0			(1.355, 2.609)
Sore throat						
Yes	415 (24.4)	68.9	31.1	19.457	<0.001	1.693
No	1284 (75.6)	56.7	43.3			(1.338, 2.143)
Rhinitis						
Yes	466 (27.4)	66.5	33.5	12.490	<0.001	1.493
No	1233 (72.6)	57.1	42.9			(1.195, 1.866)
Chills						
Yes	128 (7.5)	73.4	26.6	10.885	0.001	1.956
No	1571 (92.5)	58.6	41.4			(1.305, 2.933)
Shortness of breath						
Yes	100 (5.9)	80.0	20.0	18.229	<0.001	2.848
No	1599 (94.1)	58.4	41.6			(1.727, 4.695)
Current Health Status						
Self-perceived health						
Optimal	1280 (75.3)	52.8	47.2	101.793	<0.001	0.268
Mediocre or lousy	419 (24.7)	80.7	19.3			(0.206, 0.350)
Chronic illness						
Yes	490 (28.8)	62.9	37.1	2.885	0.089	1.206
No	1209 (71.2)	58.4	41.6			(0.972, 1.497)
Currently taking medication						
Yes	465 (27.4)	65.2	34.8	7.988	0.005	1.376
No	1234 (72.63)	57.6	42.4			(1.102, 1.717)
Admitted to hospital last 14 days						
Yes	9 (0.5)	77.8	22.2	*	0.328	2.374
No	1690 (99.5)	59.6	40.4			(0.492, 11.462)
Medical care last 14 days						
Yes	90 (5.3)	74.4	25.6	8.607	0.003	2.036
No	1609 (94.7)	58.9	41.1			(1.255, 3.304)

*—Fisher’s exact test.

**Table 4:** Association between variables related to contact history and psychological distress during the pandemic.

	Total (*n* = 1699)					
		GHQ				
	*N* (%)	Yes (*n* = 1014)	No (*n* = 685)	χ^2^	*p*	Odds Ratio (Confidence Interval at the 95 Level)
Contact > 15′ < 2 m with infected person						
Yes, or doesn’t know	567 (33.4)	63.7	36.3	5.620	0.018	1.285
No	1132 (66.6)	57.7	42.3			(1.044, 1.582)
Casual contact with infected person						
Yes, or doesn’t know	657 (38.7)	63.3	36.7	5.886	0.015	1.282
No	1042 (61.3)	57.4	42.6			(1.049, 1.566)
Contact with person or material suspected of being infected						
Yes, or doesn’t know	736 (43.3)	62.5	37.5	4.285	0.038	1.230
No	963 (56.7)	57.5	42.5			(1.011, 1.498)
Infected family member						
Yes, or doesn’t know	333 (19.6)	6.6	32.4	10.703	0.001	1.524
No	1366 (80.4)	57.8	42.2			(1.183, 1.963)
Has been performed diagnostic test						
Yes	223 (13.1)	60.1	39.9	0.018	0.894	1.020
No	1476 (86.9)	59.6	40.4			(0.765, 1.359)

**Table 5:** Contrast between preventive measures and psychological distress during the pandemic.

	TOTAL (*n* = 1699)				
		GHQ			
	M (SD)	Yes	No	Statistical	*p*
Covering mouth	4.52 (0.73)	4.49 (0.74)	4.57 (0.72)	−2.310	0.021
Avoiding sharing utensils	3.96 (1.30)	3.92 (1.31)	4.01 (1.28)	−1.382	0.167
Washing hands with soap and water	4.77 (0.50)	4.75 (0.52)	4.80 (0.49)	−2.112	0.035
Washing hands with hydroalcoholic solution	4.00 (1.03)	3.97 (1.03)	4.04 (1.03)	−1.364	0.173
Washing hands immediately after coughing, touching the nose, or sneezing	4.06 (1.02)	3.96 (1.07)	4.20 (0.93)	−4.830	<0.001
Washing hands after touching potentially contaminated objects	4.62 (0.68)	4.61 (0.68)	4.63 (0.68)	−0.766	0.444
Wearing a mask regardless of the presence of symptoms	4.48 (0.93)	4.45 (0.95)	4.53 (0.91)	−1.705	0.088
Keeping at least a metre and a half distance between others	4.51 (0.71)	4.48 (0.73)	4.56 (0.68)	−2.385	0.017

Note: Likert-type answer scale from 1 (Never) to 5 (Always).

## Data Availability

All data are available within this article. All datasets could be accessed under reasonable query to the authors.
